# Physiological framework for non-invasive detection and objective nociception activity in communicative patients: a pilot case study

**DOI:** 10.3389/fphys.2025.1704303

**Published:** 2026-01-02

**Authors:** Ghada Ben Othman, Dana Copot, Bora Ayvaz, Robin De Keyser, Clara M. Ionescu

**Affiliations:** 1 Department of Electromechanics, Systems and Metal Engineering, Research Group on Dynamical Systems and Control, Ghent University, Ghent, Belgium; 2 Flanders Make, Miro Core Lab, Ghent, Belgium

**Keywords:** nociception, pain, fractional-order impedance modeling, nociceptive variability, bioimpedance measurement, subjective–objective, variability, conscious patients

## Abstract

Pain assessment in both communicative and non-communicative patients remains a major clinical challenge due to the inherently subjective nature of conventional tools such as the Numeric Rating Scale (NRS). In this study, we introduce a physiologically grounded and objective index, ΔT=TS−TD , derived from fractional-order impedance modeling of nociceptive dynamics. Here, TD represents the transduction, and TS reflects the transmission. These components are extracted non-invasively using the Anspec-Pro device, which records skin bioimpedance in real-time. A positive ΔT indicates enhanced central excitability, while a negative value suggests dominant inhibition. In a case study of postoperative patients, we show that ΔT closely follows and often precedes subjective NRS scores, with correlation coefficients reaching up to 0.86 ( p=0.002 ). We also introduce a refined index, $\Delta T_{\text{dyn}}$ , which incorporates the trend and local variability of ΔT for improved temporal alignment with reported pain. To address the very limited dataset (three patients, nineteen intervals each), we implemented a data augmentation strategy based on autoregressive modeling of ΔT and transfer-function mapping to NRS. This approach enabled the generation of synthetic trajectories per patient, thereby enriching the dataset while maintaining physiological plausibility. Analyses of the augmented data revealed consistent lead–lag patterns, correlations, and Granger causality relationships between ΔT , $\Delta T_{\text{dyn}}$ , and NRS, suggesting that ΔT may serve both as an anticipatory biomarker of nociceptive activity and as a real-time index aligned with subjective pain reporting. Overall, these results provide proof-of-concept that the Anspec-Pro device can support objective, non-invasive nociceptive tracking in clinical environments.

## 1 Introduction

Pain is an inherently subjective and personal experience, particularly in awake and aware individuals (McMahon et al., 2013; Melzack, 1999). Although patient self-report remains the most widely accepted method for assessing pain intensity and presence (Buttner et al., 2024), it is susceptible to a variety of biases. These biases include emotional states such as anxiety or fear and, more critically, the phenomenon of tissue memory, where the perception of pain may persist even in the absence of an ongoing stimulus or may be artificially elevated by prior experience (Clemens et al., 2025; Fillingim et al., 2025; Copot and Ionescu, 2018; Becker et al., 2018; Caldichoury, 2022). To simplify clinical use, clinicians typically rely on patient self-report (Numeric Rating Scale (NRS) or Visual Analog Scale (VAS)) as the gold standard for pain assessment (Kutafina et al., 2023). However, in many clinical scenarios, for example, postoperative and critical care, patients may be awake but unable to communicate (Mert, 2023). In these cases, accurate pain evaluation is difficult, raising the risk of undetected suffering or, conversely, overmedication. Inadequate analgesia can lead to harmful stress responses and delayed recovery, whereas overuse of opioids carries serious side effects and addiction risks (Tyan and Carey, 2019; De Boer et al., 2014). For example, a systematic review reported that a notable fraction of surgical patients continue to use opioids long after discharge, highlighting the consequences of suboptimal pain management (Pagé et al., 2020). The biases of subjective assessment factors highlight a clinical motivation to develop an objective, continuous pain index or nociception activity. This index is particularly suited for assessing pain in patients who are awake yet unable to communicate verbally, offering guidance for analgesic decisions.

Recognizing the need for objective pain measures, researchers have explored various physiological signals and monitoring devices. Changes in vital signs such as heart rate, blood pressure, and respiratory patterns, as well as autonomic indicators like electrodermal activity (EDA), have been correlated with nociceptive stimuli (Kong and Chon, 2024). For example, acute pain typically triggers sympathetic activation, which can increase heart rate and sweat gland activity (lowering skin impedance) (Fernandez Rojas et al., 2023). However, in practice, these responses are neither specific nor consistently reliable for pain quantification. Stress, anxiety, or medications can produce similar autonomic changes, and individual variability is high (Cowen et al., 2015). Consequently, traditional vital sign-based assessments often do not distinguish pain from other forms of physiological excitation. In the past decade, several nociception monitors have been introduced for intra-operative or sedated patients. Notable examples include the Analgesia Nociception Index (ANI), derived from heart-rate variability; electrodermal-activity (EDA) monitors such as the Skin Conductance Algesimeter (SCA); and the multiparametric Nociception Level (NoL) index (Ledowski et al., 2022; Kim et al., 2023; Fernandez Rojas et al., 2023). These nociception metrics aim to provide a numeric indicator of the patient’s nociceptive state to guide the dosing of anesthetic and analgesic. While promising in concept, their clinical adoption has been limited. A recent validation review highlights the potential of ANI and NOL indices in monitoring nociception but also highlights the necessity for continued validation to enhance their reliability and clinical utility (Shahiri et al., 2022).

For conscious patients, researchers have investigated a range of sensing modalities and algorithms to recognize pain objectively. Computer vision and machine learning approaches have been applied to detect pain from facial expressions, voice, and multimodal physiological data (Cascella et al., 2023; Leo et al., 2020; Huang et al., 2025). For example, automated facial expression recognition systems can classify pain from video images (Tan et al., 2025). and wearable sensors have been used to classify pain versus no-pain states using features like EDA, photoplethysmographic (PPG) pulse, and electromyography (Chen et al., 2021). Such approaches have shown encouraging results in controlled studies. However, their translation to routine clinical use remains limited. Many algorithmic pain assessments have been developed on healthy volunteers or controlled experimental pain stimuli, and they may not generalize to the complexity of postoperative or critical care patients. Werner et al. report that over 70% of works on automated pain detection rely on facial cues alone, an approach that is unreliable if the patient’s face is partially covered (e.g., oxygen masks) or if the patient is immobilized (Werner et al., 2019). Moreover, factors like lighting, motion artifacts, and inter-subject variability present significant challenges.

Beyond autonomic and behavioral markers, multimodal pain-monitoring research has also focused on brain-derived electrophysiological signatures. High-density EEG studies have shown that spectral features can track both stable pain states and graded transitions during analgesia or pain resurgence, for example, in phantom-limb pain suppression under neurostimulation (Kleeva et al., 2024; Misra et al., 2017). Intracranial recordings in chronic pain have further demonstrated neural biomarkers that predict self-reported pain severity over long time scales (Shirvalkar et al., 2023). These EEG- and intracranial-based approaches provide high temporal resolution access to cortical pain processing, but they typically require complex, sometimes invasive hardware and substantial signal-processing infrastructure. In contrast, our impedance-derived framework uses a simple, non-invasive peripheral measurement to summarize the integrated peripheral–spinal–autonomic response at the skin. The proposed index ΔT is therefore best viewed as a complementary, physiology-informed correlate that can in principle be computed continuously at the bedside and could, in future work, be combined with EEG-based or intracranial markers within a multimodal pain-monitoring paradigm. An alternative to feature-based methods is a modeling approach that leverages the underlying physiology of nociception. Decades of pain research have established that pain perception involves complex dynamics with both peripheral and central components. The gate control theory proposed by Melzack and Wall first conceptualized pain transmission as a modulated process with inhibitory controls in the spinal cord (McMahon et al., 2013). Building on such foundations, mathematical models of pain signaling and control were developed, from early control-system models of pain regulation to biophysical models of nerve excitation and adaptation (Ghita et al., 2022). These models highlighted key properties of the nociceptive system, such as its nonlinear response, multiple time scales of adaptation, and memory-like effects. Modern computational studies continue this line of inquiry; for example, recent work has employed multi-scale modeling to integrate processes from ion channel kinetics up to network-level modulation of pain signals (Medlock et al., 2022). A systematic review of pain models concluded that incorporating physiology can improve the interpretability of pain predictions, but also noted that many models remain theoretical or require personalization (Lang et al., 2021).

Fractional-order dynamics has emerged as a powerful tool for capturing the inherent memory and viscoelastic behavior of biological systems, including nociceptive pathways (Copot et al., 2017). In this context, our previous studies introduced the Fractional-Order Impedance Model (FOIM) as a physiologically grounded framework to describe the dynamical processes underlying pain perception (Ionescu et al., 2019; Copot and Ionescu, 2018). The FOIM models tissue and neural responses to noxious stimuli using analogies from electrical circuit theory, where the skin-electrode interface, stratum corneum, and deeper ionic pathways are represented via resistive-capacitive elements of fractional order. This approach allows for modeling changes in bioimpedance that correspond to viscoelastic and memory phenomena observed during pain transduction and transmission. The model consists of three key components: TD , denoting the transduction of physical stimuli into electrical activity at the nociceptor level; TS , denoting the spinal transmission and modulation gain; and P , reflecting cortical perception. Each term in the FOIM corresponds to a distinct physiological stage in the nociceptive cascade, and together they define a composite impedance profile sensitive to pain-induced electrical and molecular changes (Ionescu, 2020).

These impedance dynamics are measured non-invasively by the device *Anspec-Pro*, an open source bioelectric sensor designed by our group at Ghent University for nociceptive monitoring (Ionescu et al., 2021; Ionescu et al., 2024). Its performance has been evaluated and validated in multiple experimental protocols, including cold-pressor tests and pain memory paradigms, establishing its feasibility for real-time clinical use (Ghita et al., 2019a; Ghita et al., 2019b; Ghita et al., 2023a; Ghita et al., 2020). These validations confirmed its sensitivity to nociceptive activation and its feasibility for real-time application in both laboratory and perioperative settings (Ionescu et al., 2021). The device applies a controlled electrical excitation to the skin and simultaneously records voltage and current responses to extract tissue impedance in real-time. In our multiscale modeling framework (Ionescu et al., 2019), we demonstrated that these bioelectrical signals carry distinguishable signatures of nociceptive activation and memory. Building on this modeling groundwork, our recent study (Ghita et al., 2023b) applied a parametric identification procedure to estimate FOIM parameters in patients recovering from general anesthesia. That work showed how TD and TS evolve dynamically in response to noxious stimuli and pharmacological interventions, allowing real-time characterization of nociceptive dynamics in conscious patients.

In this paper, we revisit and extend our prior work by proposing ΔT=TS−TD and its dynamic variant $\Delta T_{\text{dyn}}$ as individualized indices of nociceptive excitability. Rather than relying on the complete reconstruction of FOIM parameters for interpretation, ΔT directly captures the net amplification or damping along the nociceptive pathway, offering a simplified and interpretable physiological index. A positive ΔT indicates dominance of central transmission gain, potentially signaling sensitization, while a negative ΔT reflects a predominance of peripheral damping, suggesting inhibition or analgesia. The dynamic formulation $\Delta T_{\text{dyn}}$ further incorporates local trend and variability, improving temporal alignment with reported pain perception while retaining physiological interpretability.

The aim of this study is twofold: (i) to demonstrate the physiological consistency and subject-specificity of ΔT and $\Delta T_{\text{dyn}}$ ; and (ii) to assess the feasibility of using data collected with the Anspec-Pro device as a potential candidate for objective pain assessment in critical care. Given the small sample size (three postoperative patients), this work is presented as a preliminary proof-of-concept rather than a confirmatory clinical validation. We also designed a data augmentation procedure to support the analysis. This approach combines autoregressive modeling of ΔT with a transfer-function mapping to NRS, thereby generating multiple synthetic trajectories per patient. The augmented data were used to explore temporal properties of both ΔT and $\Delta T_{\text{dyn}}$ , including lead–lag relationships and Granger causality, providing additional insights into their physiological relevance despite the small sample size.

## 2 Methods

### 2.1 Fractional-order impedance modeling and proposed index

Capturing the inherent memory and viscoelastic behavior of biological systems, including nociceptive pathways, requires modeling tools that go beyond classical integer-order dynamics. In this context, our previous research has established a robust mathematical foundation for modeling pain transduction and perception mechanisms using fractional-order calculus (Copot and Ionescu, 2018; Ionescu et al., 2019). A key outcome of this body of work is the development of the FOIM, which characterizes the dynamic electrical properties of biological tissues under nociceptive stimulation.

**FIGURE 1:**
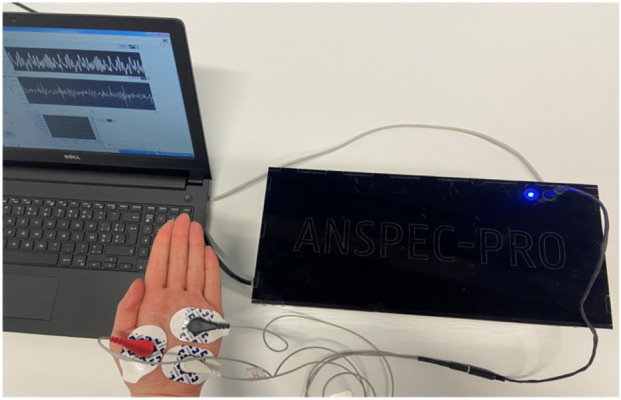
Anspec-Pro monitor with electrode placement on the palmar skin [adapted from (Neckebroek et al., 2020; Ghita et al., 2020)].

The FOIM was derived to represent the composite effects of the nociceptive processes, namely, transduction, transmission, and perception, as fractional-order elements in an electrical analog framework. These models were experimentally supported using the Anspec-Pro device (Ionescu et al., 2019), a non-invasive prototype designed for bioelectrical impedance measurement. Figure 1 shows the Anspec-Pro device and the electrode placement. The electrodes were placed on the palmar side of the non-surgical hand using the same configuration as in our previous validation work in healthy volunteers and postoperative patients (Copot and Ionescu, 2018)?

The device captures current and voltage signals at the skin surface in response to a controlled multi-sine input, enabling the derivation of complex impedance Z(s) in the frequency domain. From these signals, we identified a robust and physiologically meaningful form of FOIM expressed in Equation 1 as:

$$Z\left(s\right)=R+\frac{TD}{s^{\alpha_{1}}}+\frac{TS}{s^{\alpha_{2}}}+Ps^{\alpha_{3}},$$ (1)

where Z(s) [ Ω ) denotes the complex skin impedance in the Laplace domain, and R ( Ω ) is the baseline resistance of the tissue. The parameters TD and TS have units of [F^−1^] (inverse capacitance) and describe the transduction and transmission components of the nociceptive pathway, respectively. The term P [H] is associated with central perceptual integration and behaves analogously to an inductance. The exponents $\alpha_{1},\alpha_{2},\alpha_{3}\in \left(0,1\right)$ are dimensionless fractional orders that encode the memory and viscoelastic properties of the biological system.

The transformation from bioimpedance measurements to the physiological parameters TD and TS follows the fractional-order nociception modeling framework introduced by (Copot and Ionescu, 2018; Ionescu et al., 2019). In this framework, the measured impedance spectrum Z(jω) is decomposed into fractional-order components that represent distinct physiological processes within the nociceptive pathway. The low-frequency fractional term accounts for ionic diffusion and charge accumulation at the skin–electrode interface, which corresponds to the transduction phase ( TD ), where the noxious stimulus is converted into an electrochemical signal at the nociceptor terminals. The higher-frequency fractional term reflects the propagation of action potentials along afferent fibers, corresponding to the transmission phase ( TS ). Both TD and TS are identified through nonlinear fitting of the fractional-order impedance model to the experimental bioimpedance spectra, allowing the estimation of process-specific dynamics directly from the measured data. This formulation provides a physiologically interpretable mapping between observed impedance behavior and the underlying nociceptive mechanisms.

Physiologically, these terms correspond to distinct stages of nociceptive processing. The transduction term $TD/s^{\alpha_{1}}$ captures ionic current generation at the nociceptor level, modeled as a fractional capacitor due to the charge storage behavior across cellular membranes. The transmission term $TS/s^{\alpha_{2}}$ accounts for the propagation of spike trains through nociceptive fibers and synapses, exhibiting distributed delays and integration properties. The perceptual term $Ps^{\alpha_{3}}$ reflects (CNS) dynamics, such as cortical processing and plasticity, and behaves like a fractional inductor. The resistive term R ( Ω ) represents the high-frequency baseline impedance of the tissue and the electrode-skin interface, providing a calibration offset but not directly encoding signal dynamics.

The use of fractional-order elements is essential to reproduce the impedance spectrum of skin and neural tissue. Biological tissues such as skin and CNS (Central Nervous System) exhibit anomalous diffusion and viscoelastic behavior, leading to impedance spectra that demonstrate constant-phase behavior. These properties are well captured using fractional-order models (Copot and Ionescu, 2018; Ionescu et al., 2019). The FOIM framework offers an accurate and physiologically meaningful representation of such systems and allows for personalized modeling based on measured responses to nociceptive stimuli.

**FIGURE 2:**
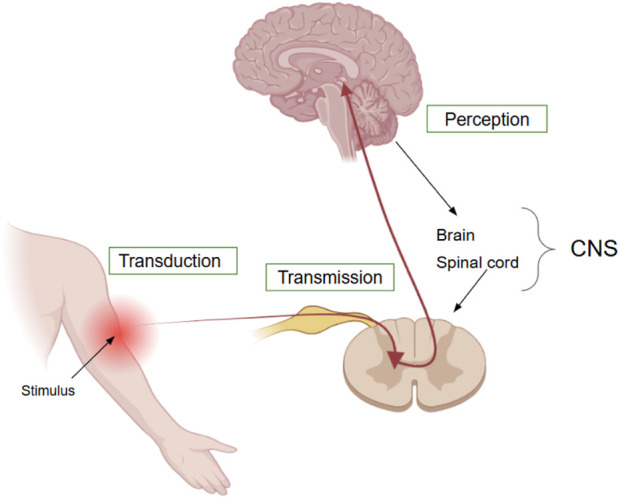
Physiological flow of nociceptive processing. A noxious stimulus is detected in the periphery (e.g., skin or muscle), where it is transduced into electrical activity by nociceptors (transduction). This signal is then transmitted via peripheral nerves and the spinal cord to the central nervous system (transmission), and finally perceived as pain in CNS (perception).

Figure 2 illustrates the anatomical and physiological sequence of nociception, comprising stimulus transduction in the periphery, signal transmission to the central nervous system (CNS), which comprises the brain and spinal cord (Yam et al., 2018). These stages correspond directly to the FOIM model components TD , TS , and P , respectively. The figure highlights the physical separation of processes: peripheral nociceptor activation (transduction), neural propagation (transmission), and cortical representation (perception), reinforcing the mechanistic mapping onto the impedance model terms.

### 2.2 Physiological basis: from nociceptor to spinal cord

The nociception process is initiated by the transduction stage occurring at the peripheral terminals of the nociceptors, where noxious stimuli (mechanical, thermal, or chemical) are translated into electromechanical signals by activating specific ion channels. This process activates transducer ion channels (e.g., Piezo2 mechanosensitive channels for incision-induced mechanical pain), allowing sodium (Na^+^) and calcium (Ca^2+^) influx that depolarizes the nociceptor membrane. If the receptor potential exceeds the threshold, voltage-gated Na^+^ channels open and an action potential is initiated. TD therefore captures the baseline ionic excitability of the nociceptor, indicating how readily a stimulus can initiate an action potential (AP). Factors that modulate TD at the nociceptor include ion channel states and inflammatory mediators: for instance, opening of potassium (K^+^) channels stabilizes the membrane and raises TD (making the neuron less excitable, acting as a “brake”), whereas inflammatory mediators (e.g., bradykinin or prostaglandins) can inhibit such K^+^ currents and augment Na^+^/Ca^2+^ influx, effectively lowering TD and making transduction easier (sensitizing the nociceptor) (McMahon et al., 2013; Choi and Hwang, 2018).

Once an action potential is generated, it propagates along the nociceptor’s axon (primary afferent) towards the spinal cord dorsal horn. This propagation (a spike train) results in neurotransmitter release at the first synaptic relay. When the spike arrives at the presynaptic terminal in the dorsal horn, the depolarization opens voltage-gated Ca^2+^ channels, causing Ca^2+^-dependent transmitter release^1^ of neurotransmitters such as glutamate^2^ and substance P^3^ into the synaptic cleft.

The next stage in the nociceptive pathway is transmission across the central synapse located in the spinal cord’s dorsal horn, often referred to as the “spinal gate.” Here, neurotransmitters released from primary afferent nociceptors (such as glutamate and substance P) activate receptors on second-order neurons. Among these, *N*-methyl-D-aspartate (NMDA) receptors^4^ play a central role, as their activation can produce a prolonged excitatory postsynaptic current. The (Ca^2+^) influx can lead to intracellular signaling cascades that strengthen the synaptic response, a phenomenon known as *central sensitization* (McMahon et al., 2013). Transmission TS quantifies the excitatory drive to trigger the postsynaptic dorsal horn neuron and transmit the pain signal to higher regions of the brain.

Importantly, the spinal cord is not merely a passive conduit; its dorsal horn serves as an active gate for incoming nociceptive signals. Inhibitory interneurons modulate the transmission strength ( TS ) of these signals through several mechanisms. For example, presynaptic release of γ -aminobutyric acid (GABA) can induce primary afferent depolarization (PAD)^5^, thereby reducing glutamate release from the primary afferent terminal.

Postsynaptically, activation of GABA and glycine receptors opens chloride ( ${\text{Cl}}^{-}$ ) channels, causing hyperpolarization or shunting inhibition of excitatory inputs. This inhibitory influence decreases neuronal excitability and lowers TS . In parallel, leak potassium (K^+^) channels^6^ continuously permit K^+^ efflux, further hyperpolarizing the postsynaptic neuron and acting as a tonic “brake” on synaptic transmission.

Conversely, TS can increase under facilitatory conditions. Intense or repetitive stimulation of afferents leads to greater activation of NMDA receptors, resulting in enhanced Ca^2+^ influx and the induction of synaptic potentiation. This form of synaptic dynamics reduces the threshold for activating postsynaptic neurons, rendering the system more responsive to subsequent stimuli. Additionally, descending excitatory projections from the brainstem, releasing facilitatory neuromodulators, can further amplify dorsal horn excitability and thereby elevate TS .

**FIGURE 3:**
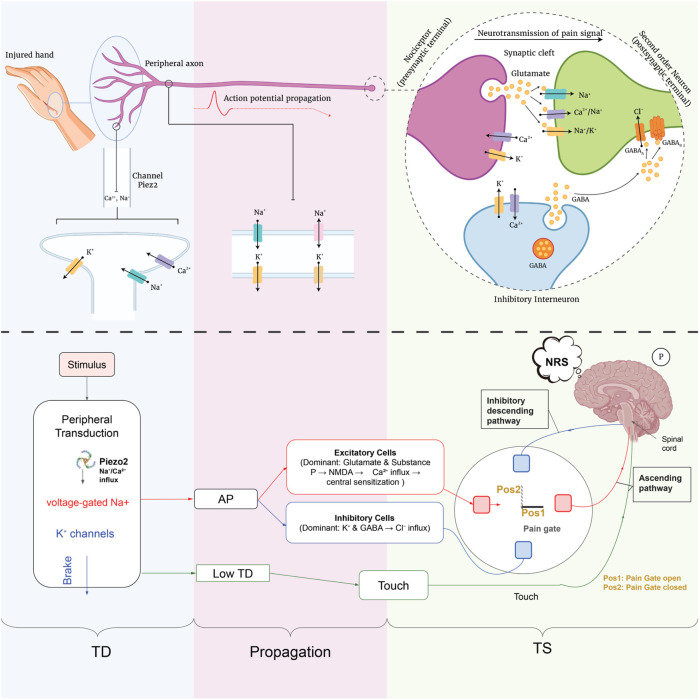
Overview of the same nociceptive pathway: an anatomical-ionic depiction (upper) and a simplified functional schematic (lower). An injured hand activates Piezo2 channels to allow Na^+^/Ca^2+^ influx (blue) against K^+^ “brake” currents, generating an action potential that travels along the peripheral axon to the dorsal-horn synapse; there, excitatory neurons release glutamate/substance P to open Na^+^/Ca^2+^ channels while inhibitory interneurons use GABA to drive Cl^−^influx and K^+^ efflux to gate the pain signal. The pain gate is open (Pos 1) when excitatory drive dominates (allowing pain transmission) or closed (Pos 2) when inhibitory drive dominates.

The complete physiological process, from peripheral transduction at the nociceptor terminals to synaptic transmission and modulation within the spinal dorsal horn, is schematically illustrated in Figure 3. The figure is structured into two complementary parts: the upper half presents an anatomical-ionic depiction, while the lower half provides a simplified functional schematic of the nociceptive process.

In the upper part, an injured peripheral tissue activates Piezo2 channels on the nociceptor membrane, allowing Na^+^ and Ca^2+^ influx, while K^+^ efflux contributes to depolarization. If the depolarization crosses the threshold, an action potential (AP) is generated and propagates along the peripheral axon. Upon reaching the central terminals in the spinal dorsal horn, this signal triggers the release of excitatory neurotransmitters such as glutamate. These activate NMDA receptors on the second-order neuron, leading to further Na^+^ and Ca^2+^ influx. In parallel, inhibitory interneurons release GABA, activating GABA_A_ receptors and resulting in Cl^−^influx that hyperpolarizes the postsynaptic membrane, inhibiting the transmission of the nociceptive signal.

In the lower part, this physiological flow is abstracted as a functional model composed of three main stages: transduction ( TD ), propagation, and synaptic transmission ( TS ). A stimulus applied to the periphery undergoes transduction via Piezo2 and voltage-gated Na^+^ channels, forming the TD component. If the transduction (TD) is insufficient (i.e., below threshold), no action potential is generated and the stimulus is perceived as touch. This is illustrated by the ‘Brake’ action of K^+^ channels opposing depolarization. If TD surpasses the threshold, an AP is triggered and propagates centrally. The signal then encounters the *pain gate*, a conceptual structure located in the dorsal horn. If excitatory interneurons dominate (e.g., glutamate and Substance P), the gate opens (Pos 1) and the signal ascends via the spinothalamic pathway, eliciting a pain response (measurable via NRS). If inhibitory interneurons dominate (e.g., K^+^ channels, GABA), the gate closes (Pos 2), blocking the nociceptive signal. A descending inhibitory pathway from the brainstem can modulate this gating mechanism, enhancing inhibition even in the presence of a nociceptive AP.

### 2.3 The TS−TD difference as an objective nociception activity

Together, the parameters TD and TS quantify two successive stages of the nociceptive pathway: peripheral *transduction* and spinal *transmission*, respectively. We interpret TD and TS as *dynamic indicators* of peripheral excitability and central synaptic gain that vary with ongoing physiological modulation. TD captures the responsiveness of the nociceptor membrane to a noxious stimulus. Its magnitude reflects the net balance of inward depolarising currents (e.g., Na^+^/Ca^2+^ influx through Piezo2, TRPV1, or voltage-gated channels) versus stabilising outward currents (e.g., leak K^+^ efflux). TS reflects the synaptic drive required for a dorsal-horn neuron to propagate incoming activity to second-order pathways^7^.

Combining both stages yields a scalar measure of the net excitability along the peripheral–spinal axis. This combination can be interpreted in terms of ionic mechanisms as follows:

$$\Delta T=\underset{\text{TS: net spinal synaptic drive}}{\underbrace{\left(\underset{1)\text{ central excitatory drive}}{\underbrace{{Ca}^{2+}}}-\underset{2)\text{ central inhibitory drive}}{\underbrace{\left(GABA+Gly+K^{+}\right)}}\right)}}-\underset{\text{TD:}3)\text{peripheral excitability}}{\underbrace{\left({Na}^{+}+{Ca}^{2+}-K^{+}\right)}},$$ (2)

In Equation 2:

Ca^2+^-triggered transmitter release (central excitatory drive);

This term reflects the amount of excitatory neurotransmitter (e.g., glutamate, substance P) released by the primary afferent terminal following Ca^2+^ influx. This release increases postsynaptic excitation in the dorsal horn.

GABA/Gly + $K^{+}$ (central inhibitory drive);

This group of inhibitory mechanisms is subtracted from the excitatory term because it reduces the overall central drive to transmit pain signals.

Na^+^/Ca^2+^ – K^+^ (peripheral excitability);

This final term represents the net ionic current at the nociceptor terminal during transduction.

We define ΔT as a composite physiological index representing the net perceived pain. Specifically, it is calculated as shown in Equation 3:

ΔT=TS−TD. (3)

Table 1 shows that a large positive ΔT indicates that spinal excitation is disproportionately strong relative to peripheral input—i.e., the system is amplifying nociceptive traffic, a signature of *central sensitization* and potentially hyperalgesia. Conversely, a negative ΔT reflects strong inhibitory gating or a high transduction requirement, consistent with analgesia or baseline conditions. ΔT<0 — Inhibition dominates: analgesic state or normal spinal gating. ΔT=0 — Balanced excitation/inhibition: baseline spinal gain. ΔT>0 — Excitation dominates: central sensitization or hyperalgesia.

**TABLE 1:** Biophysical interpretation of the three labelled terms in Equation 2.

Equation label	Increases when…	Physiological implication
1) Central excitatory drive	Presynaptic Ca^2+^ influx and vesicular transmitter release are high	Dorsal-horn synapse is *facilitated* (potentiated)
2) Central inhibitory drive	PAD, postsynaptic ${\text{Cl}}^{-}$ shunt, or leak K^+^ currents become stronger	Spinal gating is *damped*, lowering overall excitability
3) Peripheral excitability	Na^+^/Ca^2+^ entry at the nociceptor terminal is large	Nociceptor fires action potentials more readily

A high ΔT suggests a more permissive state for nociceptive signaling. Conversely, a low (or negative) ΔT implies suppression of nociceptive transmission, typically due to decreased central drive or increased peripheral inhibition (see Table 2 for an overview of possible combinations).

**TABLE 2:** Interpretation of TD, TS, and ΔT in nociceptive processing.

TD	TS	ΔT	Physiological interpretation	Expected outcome (pain perception)
High ( ↑ )	Low ( ↓ )	Very low or negative	Strong peripheral inhibition, weak central drive	No pain transmission; signal blocked at both levels
High ( ↑ )	High ( ↑ )	Low	Peripheral inhibition dominant despite strong synaptic drive	Likely no pain; central drive insufficient to overcome strong peripheral brake
Low ( ↓ )	Low ( ↓ )	Low	High peripheral excitability but minimal central relay	Weak or no pain; signal fails to reach higher centers
Low ( ↓ )	High ( ↑ )	High	Excitable periphery and strong central relay	High likelihood of pain; facilitated signal transmission
Moderate	Moderate	Moderate	Balanced peripheral and central contributions	Possible pain; context-dependent (e.g., sensitization)

In this work, we propose ΔT as a *physiological index for individualised pain management in conscious subjects*. Because TD and TS are calibrated according to the baseline impedance profile of the same person, their difference naturally compensates for the variability between subjects in the density of the nociceptor, the expression of the ion channel, and the inflammatory state. Tracking ΔT therefore, provides a patient-centric metric that continuously and objectively reports pain.

Importantly, within the FOIM framework, $T_{D}$ and $T_{S}$ are identified as deviations from each individual’s baseline impedance spectrum and expressed in arbitrary units. As a consequence, the absolute polarity of $T_{D}$ , $T_{S}$ and their difference $\Delta T=T_{S}-T_{D}$ is patient-specific and cannot be directly compared across subjects. In this proof-of-concept study we therefore interpret ΔT primarily as a within-subject contrastive index between transduction and transmission, focusing on temporal changes in ΔT for each patient rather than on absolute, cross-patient values.

### 2.4 Rationale for excluding the perceptual component P

The ΔT is designed as an objective physiological index of nociceptive activity. To preserve this objectivity, the perceptual component P , which represents the subjective experience of pain, is excluded from the model. As illustrated in Figure 3, the model focuses exclusively on the physiological nociceptive stages: transduction ( TD ), propagation, and spinal transmission ( TS ), omitting perceptual processes. The component P encompasses cognitive and emotional processes such as attention, expectation, memory, mood, and individual pain response strategies (McMahon et al., 2013). These are not merely biological signals but rather the outcome of complex neural integration within thalamo-cortical networks^8^, including areas such as the anterior cingulate cortex^9^, insula^10^, and prefrontal cortex^11^. Tu et al. (2020) As a result, P introduces a strong layer of subjectivity that varies between individuals and within the same individual depending on psychological and contextual factors (Kutafina et al., 2023). For example, emotional states such as anxiety or depression have been shown to amplify the perception of pain, whereas positive emotions or a sense of safety can significantly reduce it Hird et al. (2019). Similarly, cognitive factors such as attention and distraction have powerful modulatory effects: pain intensity ratings decrease when attention is diverted, even when the nociceptive stimulus remains unchanged (Bushnell et al., 2013).

Expectations are another major contributor to perceptual variability. Anticipating high pain often leads to an increase in pain report, a phenomenon known as expectation-induced hyperalgesia. By contrast, when individuals believe that a stimulus will be mild or non-painful, they tend to report significantly less pain, despite the physical intensity being identical. Past experiences and cultural background further shape these expectations, making it difficult to interpret pain reports in a consistent or universally applicable way. This interindividual variability has also been observed in objective physiological signals such as skin bioimpedance, where identical thermal nociceptive stimuli produced different impedance responses across participants, suggesting a modulation by personal thresholds, memory effects, or prior beliefs (Ghita et al., 2019b; Ghita et al., 2020; Ghita et al., 2023a). Recent studies using the Anspec-Pro device have demonstrated that even under standardized cold-pressor protocols, the bioimpedance signal exhibited significant time–frequency variation among individuals, some of which could be attributed to cognitive anticipation or adaptation mechanisms (Ghita et al., 2019a; Neckebroek et al., 2020). These findings underscore the limitations of relying on subjective reports for pain evaluation and further motivate the exclusion of the perceptual component P from an objective nociception activity.

As a result of its inherent subjectivity, the inclusion of the perceptual component P would compromise the aim of maintaining physiological objectivity in the ΔT index. The incorporation of P would introduce variability that is not related to the underlying physiological signal. To ensure that ΔT remains a consistent and interpretable index of nociceptive activity, and to focus specifically on the neural dimension of pain transmission, the perceptual component is excluded.

### 2.5 Electrical analogy for ΔT as an objective nociception activity

In the electrical analogy, TD can be analogized to a capacitive element in an electrical circuit. A capacitor resists sudden changes in voltage, stores energy, and absorbs input, similar to the nociceptor membrane, which accumulates ionic charge in response to mechanical, thermal, or chemical stimuli. This process is represented in the models as a fractional-order integral, similar to a capacitor. It exhibits braking behavior, meaning that a higher TD implies greater resistance to activation, reducing the propagation of the nociceptive signal.

On the other hand, the spinal cord transmission stage, reflected in TS , behaves more like an inductive component. Inductors transmit changes in current and are associated with dynamic signal propagation, especially in ladder networks that model synaptic excitation and plasticity. In the FOIM framework, TS corresponds to a fractional-order derivative, which captures the facilitative dynamics of neurotransmitter release and synaptic relay. A higher TS reflects an amplification or acceleration of signal transmission (Copot and Ionescu, 2018; Ionescu et al., 2019).

From this perspective, TD and TS operate as electrically dual components within the same nociceptive “circuit.” While one absorbs or buffers the incoming energy ( TD as capacitive resistance), the other transmits it forward with momentum ( TS as inductive drive). This aligns naturally with their opposite signs in the ΔT formulation: an increase in TD suppresses signal propagation, whereas an increase in TS promotes it.

Therefore, ΔT reflects the net excitability, regulated by the interplay of these two physiological stages. Similarly, as capacitors and inductors define temporal dynamics in physical circuits, TD and TS shape the strength of the propagation of pain signals in the nervous system.

### 2.6 Clinical analogy for ΔT as an objective nociception activity

ΔT captures the shift in dominance between these stages, offering insight into pain modulation under different conditions. A large positive ΔT indicates that the spinal cord gate amplifies nociceptive signals: an indicator of *central sensitization* (Mendell, 2014). Clinically, central sensitization involves heightened dorsal horn neuron excitability, reduced inhibition, and expansion of receptive fields. This translates to pain responses out of proportion to peripheral stimuli (McMahon et al., 2013).

A negative ΔT , indicates that peripheral requirements or inhibitory mechanisms dominate: strong spinal gating or a high nociceptor activation threshold keeps pain signals in check. Such a state corresponds to effective analgesia or normal physiological conditions where descending inhibition and local circuit damping limit pain transmission.

A key advantage of ΔT is that it provides a continuous objective assessment of pain processing, which can complement traditional subjective evaluations. Table 3 compares the ΔT metric with common patient-reported pain scales (NRS, VAS) in several practical dimensions. In particular, conventional scales such as NRS/VAS rely on the perception and report of the patient, providing only snapshot scores and no data when the patient cannot communicate. In perioperative and critical care settings, this is a serious limitation: For example, heavily sedated patients cannot give valid NRS scores. Furthermore, subjective scores are inherently variable between individuals and require the patient to internally ‘calibrate’ their 0–10 (or mild/moderate/severe) ratings.

**TABLE 3:** Conceptual mapping between ΔT ranges and expected subjective pain ratings (NRS and VAS) based on physiological reasoning.

ΔT range	Patient feeling	Expected NRS (0–10)	Expected VAS
ΔT≤0	No pain/comfort	0	No pain
ΔT≈0	Moderate pain/discomfort	1–3	Moderate pain
ΔT>0	Severe pain	3–10	Severe pain

By comparison, ΔT can be continuously calculated from physiological signals and captures the evolving balance between spinal transmission ( TS ) and peripheral transduction ( TD ) interpreted in Table 1. Together, these tables offer a dual-level perspective: Table 1 provides the mechanistic biophysical correlates of ΔT . While Table 3 illustrates how the observed physiological signatures of ΔT can be conceptually related to subjective pain ratings used in clinical practice as a theoretical reference.

### 2.7 Toward a composite index: dynamic ΔT

$\Delta T_{\text{dyn}}$ index is designed to reflect the trend of ΔT and its dynamics and variability over time. This refined index better captures the evolving nature of nociceptive response.

The construction of $\Delta T_{\text{dyn}}$ integrates three complementary components:

Baseline: We define the individual’s baseline by taking the initial value of ΔT as a personal reference point. All subsequent values are then expressed relative to this baseline, allowing us to capture individual-specific deviations over time.

Trend: Using MATLAB’s diff function (MathWorks, 2024a), we compute the first difference of the trend signal, which reflects the speed or rate of change between consecutive intervals.

Local variability: To capture short-term variations, we apply a three-point moving standard deviation to the same trend using MATLAB’s movstd function (MathWorks, 2024b). This emphasizes periods of instability or irregular transitions in the signal.

Each of these three signals ( ΔT , its speed, and its local variation) is independently normalized to the [0, 1] range. The normalized values are then summed without weights. This raw sum is finally rescaled to lie between 0 and 10, aligning the output range with the NRS pain scale (see Equation 4):

$$\Delta T_{\text{dyn}}=10\cdot \frac{1}{3}\left(\hat{\Delta T}+{\hat{\Delta T}}_{\text{speed}}+{\hat{\Delta T}}_{\text{var}}\right),$$ (4)

where $\hat{\Delta T}$ is the normalized ΔT , ${\hat{\Delta T}}_{\text{speed}}$ is the normalized speed, and ${\hat{\Delta T}}_{\text{var}}$ is the normalized variability. This dynamic version, $\Delta T_{\text{dyn}}$ , retains the physiological meaning of the original ΔT while enhancing its responsiveness to both gradual and abrupt changes.

### 2.8 Data access and reconstruction from a previous postoperative study

In this study, we revisit and build upon prior work conducted by our own research group, as reported in Ghita et al. (2023b). The original study focused on postoperative pain monitoring, where conscious and communicative adult patients recovering from general anesthesia were continuously monitored for 140 min. The goal was to assess pain through both self-reported measures and bioimpedance signals acquired with the Anspec-Pro device, a research-grade impedance monitor developed by our team at Ghent University.

The Anspec-Pro system applies a multisine voltage signal across surface electrodes positioned on the dorsum of the patient’s hand and records the resulting current and voltage responses. From these signals, the device computes the complex tissue impedance Z(f) across 29 frequencies in the range of approximately 10 Hz to 1 MHz. This approach enables continuous, non-invasive tracking of physiological changes relevant to pain assessment. In the original study, the Anspec-Pro device continuously recorded data for 140 min. Simultaneously, patients verbally reported their pain scores using the NRS, ranging from 0 (no pain) to 7 (maximum perceived pain), consistent with the limited scale used for awake and communicative patients in a postoperative setting. While the standard NRS spans 0–10, the effective range observed in PACU recordings was 0–7. This observation reflects the residual effect of anesthetic and analgesic drugs administered during surgery, which physiologically restrict the upper bound of perceived pain. Similar postoperative ranges have been reported in previous PACU studies where patients remained partially sedated or under ongoing analgesia (Sung et al., 2023).

Although the raw impedance data was not made available, the original publication presented detailed post-processed results in a graphical form. For the purpose of this study, we extracted the required data from these published figures. We extracted data from the three representative patients from the original study: patient 16, patient 18, and patient 22. Their parameter values were clearly visible and traceable in Figures 5, 6 of Ghita et al. (2023b), which show the trajectories of the FOIM parameters and the corresponding self-reported NRS scores. In these figures, the x-axis represents time intervals (in increments of 7 min), while the left and right y-axes show the model-derived FOIM parameters and the NRS scores, respectively.

The parameters of interest for this study are the TD , TS , and NRS. For TD and TS , the prior study presented boxplots at each interval for each patient. We extracted the median value from each boxplot (corresponding to the red horizontal line inside each box) across all 19 time intervals. For the NRS data, we have retrieved the score at each 7-min interval from the red square markers plotted on the right y-axis.

To ensure precision, the figures were printed on A3 format, and the extraction was performed manually by two independent team members. Each rater independently read and recorded the corresponding values, and the results were compared to assess consistency. The mean absolute deviation between the two sets of extracted values was below 2% of the plotted range for all parameters, confirming high inter-rater reliability and negligible digitization error.

In the original dataset, the first 7-min interval was reserved for model initialisation using a global optimisation routine; therefore, the analysis began from the second interval and included a total of 19 tracked intervals (133 min) for consistent comparison between model-derived parameters and patient-reported NRS scores.

The selected patients exhibit variability in both physiological and subjective pain responses, making them suitable candidates for studying the relationship between impedance-derived parameters and self-reported pain. Table 4 provides an overview of their demographic and surgical details.

**TABLE 4:** Biometric details of the three selected patients.

Patient ID	Age (yrs)	Sex/Gender	BMI (kg/m^2^)	Surgical procedure
16	35–40	F	20–24	Gynecological surgery
18	20–25	X	20–24	Urological surgery
22	30–35	F	24–30	Gynecological surgery

Abbreviations: F, female; X, transgender; BMI, body mass index.

In the original postoperative FOIM identification protocol (Ghita et al., 2023b), the full parameter set $\left[R,TD,TS,P,\alpha_{1},\alpha_{2},\alpha_{3}\right]$ was estimated for each patient and 7-min interval by nonlinear least-squares fitting of (1) to the multi-frequency impedance spectra. The fractional exponents $\alpha_{1}$ and $\alpha_{2}$ were treated as independent orders associated with the transduction and transmission branches, respectively. The resulting fits achieved low goodness-of-fit errors gof, defined in Ghita et al. (2023b) as a normalized error between measured and modelled impedance, typically in the range 0.0015–0.13 with a mean value of 0.0338, corresponding to an average fitting percentage of about 96.6% (Ghita et al., 2023b). In the present study, we did not refit the FOIM but used the published trajectories of TD and TS extracted from that published paper.

### 2.9 Data augmentation

A critical limitation of this work is the very small dataset, consisting of only three patients with nineteen intervals each. Such restricted data severely constrains both statistical analysis and model training. To overcome this limitation, we developed a data augmentation strategy that enables the generation of additional synthetic trajectories while preserving the temporal and input–output structure of the real signals.

The method proceeds in two stages. First, for each patient, the original transduction–transmission difference ΔT was modeled using an autoregressive process of order one (AR(1)). The choice of the AR(1) model was motivated by the limited number of available samples per patient (19 intervals), which prevents reliable estimation of higher-order autoregressive models (e.g., AR(2) or AR( p )) and could lead to overfitting. The model is defined as follows in Equation 5:

$$\Delta T_{k}=\phi \;\Delta T_{k-1}+\varepsilon_{k},\;\varepsilon_{k}∼N\left(0,\sigma^{2}\right),$$ (5)

where ϕ is the autoregressive coefficient estimated from that patient’s ΔT series, and $\varepsilon_{k}$ is Gaussian noise with variance $\sigma^{2}$ . This formulation captures the persistence and stochastic variability inherent to the observed ΔT dynamics. By repeatedly sampling new noise realizations, multiple synthetic ΔT trajectories are generated for the same patient.

Second, the relationship between ΔT and the reported pain scores (NRS) was approximated using a first-order difference equation of the form given in Equation 6:

$$y_{k}=-a\;y_{k-1}+b\;\Delta T_{k-1},$$ (6)

where $y_{k}$ denotes the synthetic NRS at time step k , and (a,b) are patient-specific parameters obtained by least-squares fitting to the measured data. In this framework, ΔT acts as the input and NRS as the output of a linear, first-order transfer function. Importantly, the same parameters (a,b) estimated from the real patient are reused for all of that patient’s synthetic ΔT trajectories, ensuring that the augmented data remain physiologically consistent with the original subject.

The model structure used for LSE (Least-Squares Estimation) is provided in Equation 7:

$$y\left(t\right)=\varphi^{\text{T}}\left(t\right)\theta +\varepsilon \left(t\right)$$ (7)

y(t) represents the measured output, ε(t) represents the equation errors, $\varphi^{\text{T}}\left(t\right)$ is the vector of known regressors, and θ is the vector of unknown parameters which are desired to be identified. The $\hat{\theta }$ parameter vector, which makes the loss function $V\left(\theta \right)=\frac{1}{2}\|\varepsilon \left(t\right){\|}^{2}$ minimum while ‖.‖ is the Euclidian norm vector, can be calculated using $\varphi^{\text{T}}\left(t\right)$ and measurable output vector Y as presented in Equations 8, 9:

$$\hat{\theta }={\left(\varphi^{T}\varphi \right)}^{-1}\varphi^{T}Y$$ (8)

$$\varphi =\left[\begin{array}{c} -y\left(k-1\right)\\ u\left(k-1\right) \end{array}\right]\hat{\theta }=\left[\begin{array}{c} -a\\ b \end{array}\right]$$ (9)

By repeating this two-stage process for each patient—AR(1) simulation to expand their ΔT sequences, followed by transfer-function mapping to NRS—multiple realizations can be obtained that enrich the dataset. This augmentation increases variability while respecting the original input–output dynamics. The detailed algorithmic steps and simulation procedures are provided in Algorithm 1.

Algorithm 1 Synthetic patient augmentation.

1: **Input:**Real ΔT sequence, real NRS sequence
2: Estimate AR(1) parameters ϕ and σ from ΔT

3: Estimate transfer function parameters (a,b) from (ΔT,NRS)

4: **for**each synthetic patient i=1,…,100 **do**
5:  Initialize synthetic $\Delta T^{\left(i\right)}\left(1\right)\leftarrow \Delta T\left(1\right)$

6:  **for** k=2 to N **do**
7:   Generate $\Delta T^{\left(i\right)}\left(k\right)\leftarrow \phi \Delta T^{\left(i\right)}\left(k-1\right)+\varepsilon_{k}$ , $\varepsilon_{k}∼N\left(0,\sigma^{2}\right)$

8:  **end for**
9:  Initialize synthetic NRS $y^{\left(i\right)}\left(1\right)\leftarrow \max\left(\text{NRS}\right)$

10:  **for** k=2 to N **do**
11: $y^{\left(i\right)}\left(k\right)\leftarrow -a\;y^{\left(i\right)}\left(k-1\right)+b\;\Delta T^{\left(i\right)}\left(k-1\right)$

12:   If $y^{\left(i\right)}\left(k\right)<0$ , then $y^{\left(i\right)}\left(k\right)\leftarrow 0$ (enforce non-negativity)
13:  **end for**
14: **end for**
15: **Output:**100 synthetic ΔT and NRS trajectories per real patient

## 3 Results

**FIGURE 4:**
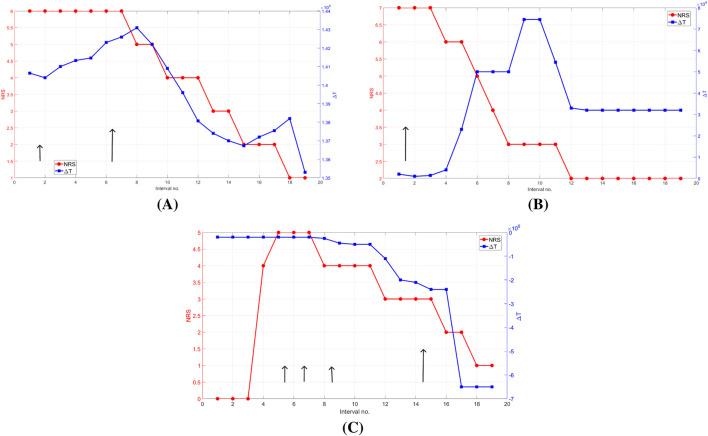
Comparison of ΔT (blue, right axis) and NRS (red, left axis) across 19 postoperative intervals (each interval is 7 min) in real patients. Data are from actual recordings. Short black arrows indicate light opioid administration (e.g., Piritramide); long arrows denote heavier opioid doses (e.g., Tramadol). **(A)** Patient 22; **(B)** Patient 18; **(C)** Patient 16.

In this section, we present a comparison of ΔT and NRS scores for the three patients (Figure 4). Each trajectory extends over 19 equal time intervals.

In patient 16 (Figure 4C), the NRS increases from 0 to 5 by interval 5, while ΔT remains nearly flat and negative until interval 8. At that point, it begins to decline, one interval after the NRS starts decreasing, indicating a slight lag in the FOIM signal. However, following the heavy opioid dose at interval 15, ΔT drops sharply and becomes strongly negative, reflecting a rapid physiological shift. This negative swing is immediate and much more pronounced than the gradual decline in NRS, which only starts decreasing two intervals later. In this final phase (intervals 15–19), ΔT clearly leads the subjective score and shows a larger and faster dynamic, capturing the analgesic effect more promptly than the NRS.

In Figure 4B, patient 18 starts with a consistently high NRS of seven and a low ΔT value around $2\times 10^{3}$ . A heavy opioid dose is administered at interval 1, yet the NRS remains high and stable until interval 3. At that point, ΔT rises sharply, and the NRS begins to decline at the same interval. This simultaneous response contrasts with the earlier lack of change, suggesting a delayed effect of the initial dose or variability in sensitivity. ΔT reaches a peak near $7.5\times 10^{3}$ before dropping rapidly starting at interval 9. It stabilizes around $3\times 10^{3}$ from interval 13 onward, while the NRS continues to decrease gradually until it reaches zero at interval 12.

This pattern shows a mismatch between the physiological index and the reported pain. The overshooting behavior of ΔT , not aligned with any new drug input, may reflect variations or noise in the spinal dynamics.

In patient 22 (Figure 4A), ΔT gradually increases from interval 1 to 8, while the NRS remains constant at 6. The ΔT peak aligns precisely with the first drop in NRS at interval 8, suggesting a synchronous physiological and subjective response at that moment. From intervals 9 to 13, both signals decrease together. However, ΔT begins its decline slightly before the second drop in NRS at interval 10, suggesting a short lead of ΔT . Interestingly, once the NRS stabilizes at a low level (around interval 13 onward), ΔT continues to show minor variations. These variations may reflect an ongoing modulation in the spinal transmission system that is not captured yet by the patient’s conscious perception.

Across all three cases, ΔT consistently exhibits a larger relative amplitude and faster response to drug than the NRS. The variability in ΔT (especially in patient 22 at low pain levels) suggests that ΔT captures the dynamics of spinal transmission before NRS, underscoring the potential of ΔT as a fast and objective biomarker of pain.

### 3.1 Correlation between ΔT and NRS scores

To evaluate the relationship between ΔT and the subjective NRS, Pearson correlation coefficients ( r ) and corresponding p -values were computed for each patient. Table 5 summarizes the statistical results for the three patients (16, 18, and 22).

**TABLE 5:** Pearson correlation magnitude ( |r| ), 95% confidence interval (CI), and p -values between ΔT and NRS scores for each patient (n = 19 intervals per patient).

Patient ID	|r|	95% CI for |r|	p -value
22	0.86	[0.67, 0.95]	$2.09\times 10^{-6}$
16	0.38	[0.09, 0.71]	0.1083
18	0.57	[0.16, 0.82]	0.0103

Correlation coefficients and associated p-values in this section are computed exclusively from the original 19 postoperative intervals per patient. Among the three cases, patient 22 shows the strongest correlation ( r=0.86 , 95% CI [0.67, 0.95], p<0.001 ), indicating a tight and statistically significant alignment between ΔT and NRS scores. This strong association suggests that ΔT effectively tracks the patient’s pain evolution, supporting its value as an objective physiological marker of nociceptive activity. Patient 16 also demonstrates a statistically significant moderate correlation ( r=0.57 , 95% CI [0.16, 0.82], p=0.010 ), confirming that ΔT reflects clinically relevant pain dynamics. Although some temporal offset is visible in Figure 4C, the overall strength of the association highlights the capacity of ΔT to anticipate or complement patient-reported pain trends.

Conversely, patient 18 shows a weak and non-significant correlation ( r=0.38 , 95% CI [0.09, 0.71], p=0.11 ), suggesting limited synchrony between ΔT and NRS scores in this individual. This deviation may reflect case-specific variability, inconsistent pain reporting, or non-linear physiological dynamics. It also highlights the need for further investigation into the conditions under which ΔT most reliably captures nociceptive changes, especially in cases where subjective and physiological responses show mismatch.

Overall, these results confirm that ΔT can reflect nociceptive dynamics with high fidelity in some cases (e.g., patients 22 and 16), while in others it may detect complementary or delayed responses not fully captured by the NRS alone. However, given the limited number of intervals available per patient (n = 19), these correlation estimates and their confidence intervals should be interpreted as exploratory rather than definitive evidence of effect size or generalizable performance.

### 3.2 Further observations

Patient 16 exhibited an unexpected sign inversion. Throughout the recording, ΔT remained negative, whereas the model presented in this paper links negative values of ΔT to an NRS close to zero. Nevertheless, the recorded NRS ranged from 0 to 5. The two curves were still significantly correlated ( r=0.57,p=0.010 ), indicating that the index followed the patient’s reported pain. This observation necessitates further investigation and calibration in future studies. Consistent with this interpretation, all analyses in this work focus on within-patient dynamics: ΔT was always computed as $T_{S}-T_{D}$ using the identified FOIM parameters, and its physiological relevance was assessed through patient-specific correlations, lead–lag relationships, and the rescaled dynamic index $\Delta T_{\text{dyn}}$ . In other words, the clinically meaningful information in this initial case series lies in how ΔT changes over time within a given patient, rather than in its absolute polarity across patients.

Nociceptive responses vary widely between individuals, making inter-patient variability a key challenge in pain modeling (Fernandez Rojas et al., 2023). This variability reflects differences in physiological factors such as nociceptor density, ion channel behavior, and inflammatory state. Several modeling studies support this observation. For example, Table 2 in our prior work (Ghita et al., 2023b) shows that the estimated parameters TD and TS exhibit large dispersion across patients. Their standard deviations are often comparable to or greater than their means, confirming that this variability is not due to noise but is a characteristic of the population. Additional evidence for such variability has been reported in bioimpedance studies, where thermal-induced nociception protocols revealed patient-specific patterns in impedance evolution. In particular, these studies observed that some individuals exhibited persistent shifts in impedance even after stimulus removal: an effect interpreted as residual pain or thermal memory (Ghita et al., 2019b; Ghita et al., 2019a; Ghita et al., 2023a).

Similar trends appear in studies using other modalities. EEG measurements reported by Tiemann et al. (2018) show broad differences in the latencies of pain-related evoked potentials between individuals, further supporting the presence of high inter-patient variability in nociceptive processing. The systematic review by Lang et al. (2021) highlights that most mathematical pain models require individual calibration, since average parameters fail to capture patient-specific dynamics.

In addition to differences in magnitude, TD and TS often have opposite signs, as discussed in a previous section. As shown in Ghita et al. (2023b), many patients present with negative TD and positive TS , while others show the reverse. This observation supports the use of ΔT as a contrastive index, preserving the directional relationship between TD and TS . However, it also implies that averaging these parameters between patients could cancel out the interpretability of the sign ΔT .

Moreover, in the FOIM (1) presents the exponents $\alpha_{1}$ and $\alpha_{2}$ as the tissue memory at the transduction and transmission stages, respectively. The experimental fittings show that within a single patient, $\alpha_{1}$ and $\alpha_{2}$ converge to almost equal values (Ghita et al., 2023b; McMahon et al., 2013). This similarity is reasonable because both peripheral and spinal tissues undergo comparable viscoelastic adaptation and long-term sensitisation. Setting the exponents equal therefore, as expressed in Equation 10, reflects a shared memory kernel:

$$\alpha_{1}=\alpha_{2}=\alpha \;\Rightarrow \;\frac{TD}{s^{\alpha }}+\frac{TS}{s^{\alpha }}=\frac{TD+TS}{s^{\alpha }}.$$ (10)

Notice that when $\alpha_{1}$ and $\alpha_{2}$ converge to a single α , the two fractional terms merge TD and TS become mathematically inseparable. Under this condition, the sign of the difference ΔT=TS−TD no longer influences the model response.

### 3.3 Results of the dynamic ΔT

**FIGURE 5:**
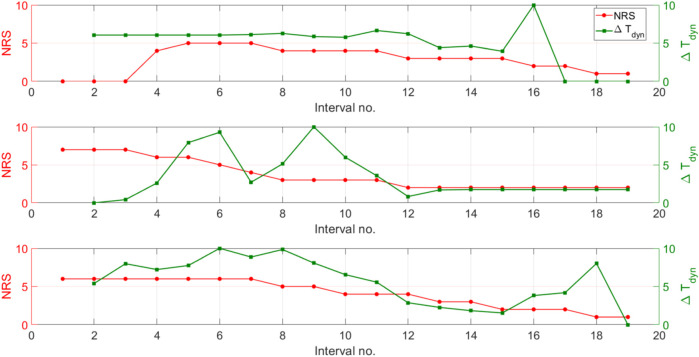
Comparison between NRS (red) and the proposed dynamic index $\Delta T_{\text{dyn}}$ (green) for patients 16 (top), 18 (middle), and 22 (bottom). The right axis scales $\Delta T_{\text{dyn}}$ between 0 and 10; NRS values are plotted on the left axis. All trajectories shown in this figure correspond to original, non-augmented patient data.

Direct interpretation of ΔT is limited by sign changes and patient variability. To address these issues, we introduce a composite index, $\Delta T_{\text{dyn}}$ . Figure 5 displays the dynamic index $\Delta T_{\text{dyn}}$ alongside the self-reported NRS values for three patients. Compared to ΔT , the new index incorporates local dynamics, namely, trend (instantaneous speed) and local variability of ΔT , which together improve its sensitivity to changes in pain.

Across all three patients, $\Delta T_{\text{dyn}}$ broadly follows the temporal evolution of the NRS, capturing key transitions in reported pain. In patients 16 and 22, the dynamic index often leads the NRS, showing slight increases or drops before the corresponding changes in self-reported scores, suggesting its potential to anticipate changes in pain. In contrast, for patient 18, some features of $\Delta T_{\text{dyn}}$ lag behind the NRS, indicating delayed physiological reflection of reported pain. These lead–lag dynamics underline the index’s ability to track the magnitude and the temporal profile of pain experience.

### 3.4 Interpretation of augmented data

**FIGURE 6:**
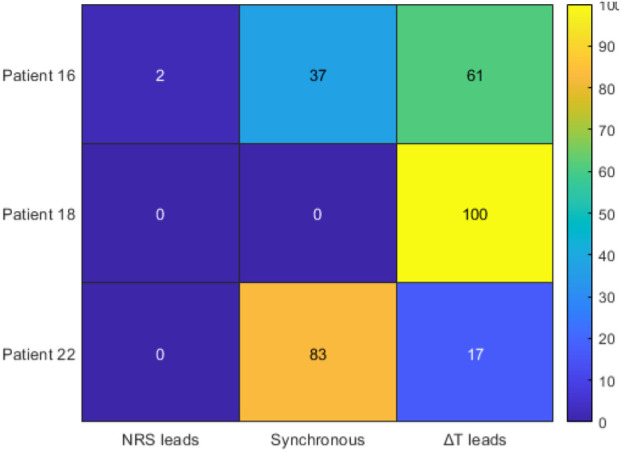
Lead–lag distribution for synthetic ΔT .

**FIGURE 7:**
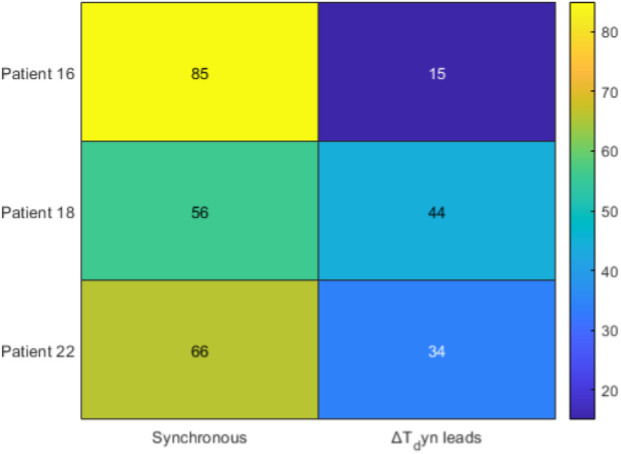
Lead–lag distribution for synthetic $\Delta T_{\text{dyn}}$ .

**FIGURE 8:**
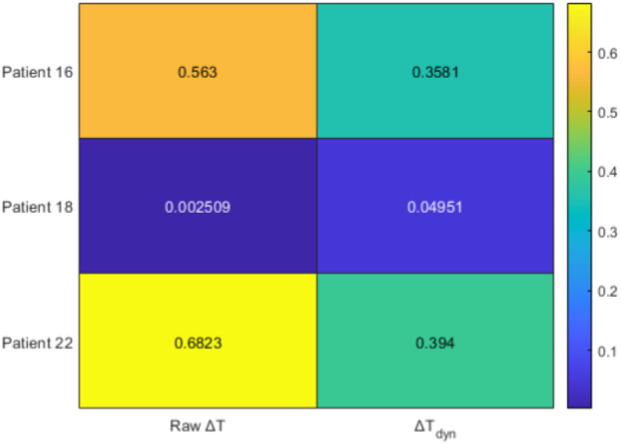
Mean Spearman correlations between augmented trajectories and NRS.

**FIGURE 9:**
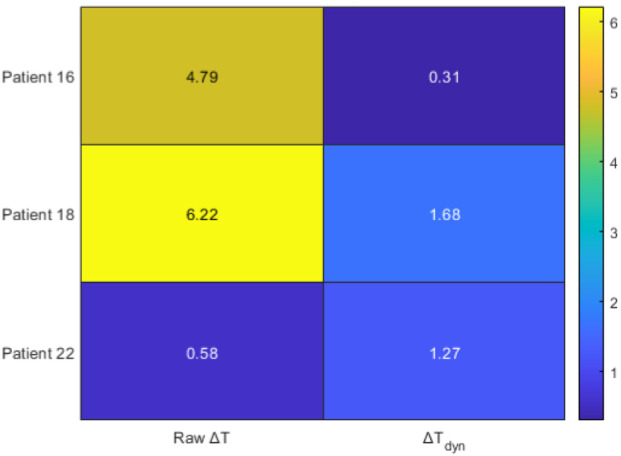
Mean lag values across patients.

To further explore the temporal dynamics between ΔT and subjective pain scores, we applied the augmentation strategy to generate one hundred synthetic trajectories per patient. We then analyzed correlations, lead–lag patterns, synchrony, and Granger causality between ΔT , $\Delta T_{\text{dyn}}$ , and NRS scores. Lead–lag patterns were quantified using cross-correlation functions, where the lag corresponding to the maximum absolute correlation determined whether ΔT led, lagged, or was synchronous with NRS. In addition, Granger causality tests were performed in MATLAB by comparing autoregressive models with and without ΔT terms. Figures 6–9 illustrate the main outcomes.

Figure 6 shows that for Patient 16, ΔT leads NRS in 71% of cases, for Patient 18 it always leads (100%), and for Patient 22 it is mostly synchronous (74%) with ΔT leading in the remaining 26%. This indicates that ΔT frequently precedes subjective pain reporting, consistent with the physiological expectation that nociceptive transmission occurs faster than conscious perception. When applying the dynamic formulation (Figure 7), the pattern changes: Patient 16 becomes predominantly synchronous (86%), Patient 18 shows mixed outcomes with approximately 56% synchronous and 44% $\Delta T_{\text{dyn}}$ leading, while Patient 22 is nearly balanced (51% synchronous, 49% leading). As a result, compared to ΔT , the dynamic index reduces systematic leads and better aligns with subjective pain reports. This shift is further reflected in the correlations (Figure 8), where ΔT shows stronger associations with NRS (Patient 16: r=0.62 , Patient 22: r=0.52 ) than $\Delta T_{\text{dyn}}$ (Patient 16: r=0.41 , Patient 22: r=0.32 ), while Patient 18 remains poorly correlated in both cases ( r≈0.08 ). Finally, the lag analysis (Figure 9) confirms that ΔT systematically leads NRS by about six samples in Patients 16 and 18 and by one sample in Patient 22, whereas $\Delta T_{\text{dyn}}$ reduces this delay to within 0–2 samples across all patients.

The analysis demonstrates two complementary aspects of ΔT . On one hand, ΔT exhibits stronger correlations with NRS and consistently leads the reported scores, reflecting the fact that spinal transmission dynamics precede conscious perception. This anticipatory property suggests that ΔT can detect nociceptive activation earlier than patients can verbally report it, supporting its use as a predictive biomarker of nociception. On the other hand, the dynamic variant $\Delta T_{\text{dyn}}$ trades off correlation strength for temporal alignment, reducing systematic leads and providing a more synchronous index. This makes $\Delta T_{\text{dyn}}$ better suited for real-time nociception monitoring applications. Together, these findings confirm the physiological relevance of ΔT : it reflects nociceptive activity and captures whether spinal responses anticipate or align with conscious pain reporting.

Table 6 presents the Granger causality test outcomes. For Patient 18, both models show highly significant causality (very large F -statistics and p<0.001 ), indicating that changes in ΔT robustly precede changes in pain scores. In contrast, Patient 16 shows very large F -values but non-significant p -values ( p>0.05 ), which suggests statistical instability due to the short time series. These results therefore indicate strong apparent effect sizes but insufficient reliability, and any implication of predictive validity should be avoided. Patient 22 does not exhibit significant Granger causality ( p>0.6 for both raw and dynamic forms), consistent with the weaker coupling observed in correlation and lead–lag analyses. Overall, the results suggest that while ΔT may carry predictive information in some patients (e.g., Patient 18), such findings should be interpreted cautiously, as model reliability varies considerably across individuals.

**TABLE 6:** Mean Granger causality statistics across 100 synthetic realizations per patient, comparing raw ΔT and $\Delta T_{\text{dyn}}$ as predictors of NRS.

Patient	Count	mean_F_gc_raw	mean_p_gc_raw	mean_F_gc_dyn	mean_p_gc_dyn
Patient 16	100	$1.93\times 10^{28}$	0.125	37.47	0.148
Patient 18	100	$6.81\times 10^{27}$	0.000	42.54	0.0002
Patient 22	100	0.60	0.660	0.76	0.607

## 4 Discussion

This research examines the feasibility of using ΔT as a novel, patient-specific, and objective measure of nociception activity. It applies to both communicative and non-communicative aware patients, based on physiological models. The hypothesis is that pain intensity can be objectively quantified using a fractional-order model parameter ( ΔT ). This parameter is derived from the dynamics of transduction and transmission in the nociceptive pathway. To test this, we analyzed postoperative data from three patients, recorded using the Anspec-Pro device, which measures electrical bioimpedance signals associated with nociceptive processing. The key finding is that ΔT is strongly correlated with self-reported pain scores (NRS) of patients in most cases. Two out of three patient profiles exhibited a moderate positive correlation between ΔT and NRS (Pearson’s r>0.54 , p<0.001 ). This indicates that increases or decreases in pain were mirrored by corresponding changes in ΔT . Furthermore, ΔT changes were often interpretable in terms of underlying physiological transitions captured by the Anspec-Pro. These transitions include variations in nociceptor excitability and spinal transmission dynamics. Notably, ΔT frequently anticipated shifts in NRS values, highlighting its potential as a leading indicator of nociceptive changes. This latency advantage suggests that ΔT can detect changes in the pain pathway before they are verbally reported, an observation that warrants further investigation in the context of real-time pain monitoring.

The lead–lag analysis of both real and augmented data provides important physiological insight. Cross-correlation patterns consistently showed that raw ΔT tends to lead NRS, particularly in Patients 16 and 18. This reflects that nociceptive transmission changes often precede conscious pain reporting. This anticipatory property underscores the potential of ΔT as an early-warning signal of nociceptive activation. In contrast, the dynamic index $\Delta T_{\text{dyn}}$ reduced systematic leads and achieved greater synchrony with NRS, aligning more closely with the subjective timeline of pain perception. This trade-off suggests complementary applications: raw ΔT as a predictor of upcoming nociception, and $\Delta T_{\text{dyn}}$ as a real-time monitoring tool. Together, these findings suggest the potential clinical utility of the proposed indices for anticipatory and synchronous pain assessment.

A major advantage of the ΔT index is its clear physiological interpretability and novelty in the context of pain monitoring. ΔT corresponds to a physiological state along the pain pathway. A high ΔT ( TS · TD ) would indicate that the central nervous system is providing significant amplification relative to peripheral input. In other words, the patient’s spinal cord is sensitized to transmit the nociceptive signals. In contrast, a low ΔT implies dominant damping, which means that the spinal gating mechanisms blunt the incoming signals (or the peripheral input itself is low relative to the central tone of the baseline). By decomposing pain into these components, the ΔT index provides insight into where and how pain is being modulated in the body. This physiological grounding sets ΔT apart from prior pain indices and makes it especially useful in complex clinical scenarios.

A further contribution of this work is the introduction of a data augmentation strategy tailored to the limited dataset of three patients. By combining autoregressive modeling of ΔT with a patient-specific transfer-function mapping to NRS, we generated one hundred synthetic trajectories per subject. This approach increased variability while preserving the original input–output dynamics, enabling more robust analysis of temporal relationships. Although effective as a proof of concept, the augmentation procedure remains simplistic: it assumes linear autoregressive dynamics and a first-order transfer mapping. Future work could extend this framework by exploring non-linear mappings, multi-input autoregressive models, or population-level augmentation strategies, thereby improving the fidelity and generalizability of the synthetic data.

In Algorithm 1, the synthetic NRS trajectory was initialised at the maximal recorded NRS value for each patient. This choice is clinically motivated: immediately after surgery, residual anaesthetic and analgesic drugs can transiently suppress the patient’s pain perception, resulting in abnormally low or zero NRS values at the start of the monitoring period. For instance, in patient 16, the first recorded NRS was close to zero due to the effect of intraoperative medication. Using such a value as the initial condition would produce an artificial rise in the simulated pain trajectory and distort its correspondence with the transfer-function dynamics. By contrast, initialising at the maximal NRS reflects the physiologically meaningful onset of postoperative pain, after the anaesthetic effect has dissipated, and aligns with the expected clinical pattern of gradually decreasing pain following analgesic administration.

From a clinical point of view, the design of ΔT also means that it remains valid regardless of the patient’s communication status. In communicative patients (for example, those awake in the post-anesthesia care unit), ΔT can be recorded alongside NRS or verbal pain scores, serving as a cross-check between subjective perception and the processing of underlying physiological pain. In non-communicative patients (e.g., sedated or delirious critical care patients, or intraoperative patients under general anesthesia), subjective pain assessment tools fail because the patient cannot report their pain. In these cases, ΔT offers a continuously updated objective measure that does not rely on patient input.

Compared to other objective pain indices such as the NOL index, which combines multiple physiological signals and Artificial intelligence, ΔT is simpler and easier to interpret. It relies only on bioimpedance signals recorded by the Anspec-Pro device. It directly reflects changes in the nociceptive pathway, capturing both peripheral input and spinal transmission. This makes ΔT a physiologically grounded and easily accessible tool for real-time pain assessment.

It is important to note that although ΔT is statistically compared with the subjective NRS for validation, the index itself does not include the perceptual component P of the FOIM. This design choice ensures that ΔT remains a purely physiological descriptor of nociceptive activity—limited to the measurable ( TD ) and ( TS ) processes—while NRS serves only as an external reference of perceived pain. The correlation between ΔT and NRS thus confirms physiological alignment without implying that perception is modeled within ΔT .

While the original ΔT index captures fundamental nociceptive dynamics, it is sensitive to signal variability and model underdetermination. Both TD and TS are identified from noisy bioimpedance data, and the system of equations derived from the FOIM model is underdetermined (Ionescu et al., 2019). This is especially problematic when the estimated fractional orders $\alpha_{1}\approx \alpha_{2}$ , reducing the model’s ability to separate the transduction and transmission phases. This limitation was observed in patient 16, where ΔT remained negative throughout the recording period, even as the NRS steadily increased.

To address these challenges, we introduce $\Delta T_{\text{dyn}}$ , a dynamic variant that combines the trend and variability of ΔT . This refinement preserves physiological interpretability while improving responsiveness and robustness.

$\Delta T_{\text{dyn}}$ follows the trend of NRS and often anticipates pain score changes in communicative patients (e.g., patients 16 and 22). However, patient 18 presents a notable exception: while NRS decreases steadily, $\Delta T_{\text{dyn}}$ rises sharply before stabilizing. This inverse pattern may reflect dominant spinal inhibition, distorted estimation due to overlapping dynamics, or local electrode artifacts. Such cases highlight the need to interpret $\Delta T_{\text{dyn}}$ within a broader physiological context.

In addition to acute pain monitoring, the index $\Delta T_{\text{dyn}}$ is valuable for long-term observation. It combines the level, speed, and variability of pain-related dynamics, allowing it to detect both stable trends and sudden changes. As a result, it can help identify slow increases in pain or changes in drug response. Normalization to the baseline of each patient makes it possible to track meaningful changes over time. In chronic or disease-related pain, where the pain pathway can change over days or weeks, $\Delta T_{\text{dyn}}$ could provide an objective signal of increased spinal sensitivity or reduced inhibition. This signal would complement the remote follow-up procedure demonstrated in trials based on Internet cognitive behavioral therapy (De Boer et al., 2014). It may also be helpful when patients cannot communicate regularly, making the index suitable for use in extended care.

From a practical standpoint, the computation of ΔT is compatible with real-time monitoring. In previous work with the same Anspec-Pro device, FOIM parameters were identified recursively with minute-scale updates during PACU monitoring, and were proposed as inputs for closed-loop analgesia controllers. Building on this, ΔT and $\Delta T_{\text{dyn}}$ only require algebraic combination and light temporal smoothing of the already-available $T_{D}$ and $T_{S}$ trajectories. This makes their implementation comparable in complexity to current nociception indices, while providing a more direct link to underlying nociceptive mechanisms. 

### 4.1 Analgesic modulation of transduction and transmission

An important aspect of this study was demonstrating that ΔT is sensitive to common pain interventions and their distinct mechanisms of action. Clinical pain management employs interventions at different levels of the pain pathway, mainly analgesics such as opioids and regional anesthetic blocks. These interventions have well-known, but differing, physiological effects on nociceptive transduction and transmission, and the results indicate that the ΔT index captures these effects in real-time. This provides a mechanistic justification for using ΔT as a pain metric. Opioid analgesics (such as morphine, fentanyl, or remifentanil), which are systemic or central analgesics, primarily act at the level of the spinal cord and brainstem to attenuate pain transmission. Opioids connect to opioid receptors that are densely expressed on the primary afferent terminals in the dorsal horn of the spinal cord and in the pain-modulating centers of the brain. Consequently, opioids significantly reduce synaptic transmission in the spinal cord.

In terms of FOIM model, these pharmacological actions predominantly reduce the TS parameter (diminishing central transmission and amplification), while leaving TD (peripheral transduction sensitivity) largely unaffected. Consistent with this, our data showed that after opioid administration (in both real and simulated patients), the ΔT value tended to drop simultaneously with NRS reduction, reflecting the fact that the spinal component of nociception was being pharmacologically dampened while the peripheral input remained the same or only slowly changing. Importantly, ΔT often began to fall shortly after opioid delivery, even before patients reported feeling less pain, reinforcing that ΔT dynamically tracks the physiological impact of analgesics in real-time.

### 4.2 Pharmacological modulation of nociceptive dynamics

Patient 18 presents a distinct profile compared to the other two cases. Both the ΔT and $\Delta T_{\text{dyn}}$ indices showed rising or unstable trajectories, despite a progressive decrease in the reported NRS. Notably, this patient received only a one analgesic, unlike patients 16 and 22 who were administered stronger opioids. The persistence of elevated ΔT values suggests that nociceptive transmission at the spinal level ( TS ) remained insufficiently inhibited, potentially reflecting a physiological state of ongoing pain that was not fully suppressed by the limited pharmacological input.

This observation is particularly informative when extrapolated to the intraoperative setting. During general anesthesia, patients are unable to communicate, and therefore rely entirely on physiological signals to guide pain management. In such settings, drugs like remifentanil and propofol are used because of their potent effects on spinal and cortical inhibition. If, however, ΔT exhibits an increasing trend even under these agents, it may indicate that spinal transmission is not being adequately suppressed, pointing to a potential failure in inhibitory control mechanisms. This would suggest a need to evaluate the ongoing drug effect, especially in closed-loop or adaptive systems where analgesic dosing must be continuously optimized.

Real-time tracking of physiological indices like $\Delta T_{\text{dyn}}$ could therefore serve as a sensitive indicator of analgesic efficacy, both in conscious recovery and during full unconsciousness (general anesthesia). Integrating such feedback into model-based drug delivery systems could enhance the safety and precision of intraoperative analgesia. An increase in ΔT under strong anesthetic agents may signal that spinal inhibition is insufficient. This could prompt timely adjustment of remifentanil or propofol doses.

### 4.3 Limitations and perspectives

This study has several limitations due to its case study design, which should be taken into account when interpreting the results.

The first limitation arises from the size of the dataset. Only three patients were analyzed, all undergoing the same type of surgery and observed in the same context (postoperative). As a consequence, all reported correlations and confidence intervals should be viewed as preliminary, exploratory estimates rather than precise or generalizable effect sizes, and the available sample size does not allow us to achieve conventional statistical power (1−β) for biomarker validation. In this sense, the correlation analyses are intended as proof-of-concept descriptors rather than powered confirmatory tests. Likewise, the AR(1)-based data augmentation described in Section 2.9 does not increase the number of independent observations and therefore does not improve formal test power; it is used solely to explore temporal dynamics under the same subject-specific ΔT –NRS relationship. This design choice was intentional, as the use of the same surgical procedure helped to ensure a consistent source of nociception and minimized confounding variability. However, this limited scope restricts the generalizability of the findings. Future studies should include larger and more diverse patient cohorts, encompassing various types of surgery, age groups, comorbidities, and pain mechanisms (e.g., incisional, visceral, neuropathic).

Second, although impedance-derived FOIM parameters were used, continuous raw impedance signals were not available. ΔT was reconstructed from discrete, manually derived values rather than computed in real-time. This non-automated approach may miss transient dynamics and introduces potential manual bias. Incorporating continuous data streams and fully automated pipelines is essential for clinical translation, especially in time-critical environments such as the operating room. In future work, integrating physiologically digital twins could help test the robustness of ΔT under controlled perturbations and simulated pharmacological profiles.

Another important limitation relates to the physiological interpretation of the FOIM parameters TD and TS . Their labelling as “transduction” and “transmission” follows from a multi-scale, biophysically motivated modelling framework, but this mapping is indirect and cannot be empirically verified from skin-impedance data alone. In practice, the measured bioimpedance reflects a mixture of local skin properties (e.g., sweat gland activity, hydration, microvascular tone) and systemic sympathetic drive, so ΔT should presently be regarded as a physiology-informed autonomic correlate of nociceptive activity rather than a direct quantitative measure of spinal excitability. Moreover, the current dataset did not include concurrent GSR, EMG or EEG recordings, preventing any multimodal comparison with established electrophysiological or autonomic pain markers. Future studies combining Anspec-Pro measurements with such modalities will be essential to validate and refine the mechanistic interpretation of ΔT and $\Delta T_{\text{dyn}}$ . Moreover, all measurements in this pilot were acquired at a single distal site, namely, the palmar skin of the non-surgical hand, which provides a global autonomic correlate of nociceptive activity but no spatially localized information about the surgical field; future studies should systematically compare this palmar configuration with alternative or additional electrode locations.

A further limitation is that the observational dataset used in this pilot study did not include concurrent recordings from established nociception monitoring systems such as ANI or NoL. Only Anspec-Pro bioimpedance measurements and subjective NRS scores were available, which precluded any direct comparison between ΔT (or $\Delta T_{\text{dyn}}$ ) and existing indices. As a result, the added value of ΔT is inferred here from its physiological grounding and its temporal relationship with NRS, rather than from formal superiority metrics. Future prospective studies should acquire ΔT in parallel with ANI, NoL and SCA to quantify incremental clinical utility under the same analgesic interventions. Finally, the study was confined to a specific setting: postoperative pain. The validity of ΔT in other clinical contexts, such as chronic pain management or during general anesthesia, remains to be explored. For example, under total intravenous anesthesia with agents like propofol and remifentanil, spinal nociceptive transmission may be pharmacologically suppressed to varying degrees. Understanding how ΔT behaves under such conditions is critical for assessing its utility as a physiological feedback marker in intraoperative monitoring or sedation control.

## 5 Conclusion

This study presents a proof-of-concept for ΔT as a possible novel, objective index of nociceptive activity derived from fractional-order bioimpedance modeling. Identified using the Anspec-Pro device, this index captures the physiological dynamics of nociceptive transduction and transmission, enabling individualized pain tracking without reliance on subjective input. In a three-patient pilot case study, ΔT showed temporal association with patient-reported NRS scores and often preceded reported pain changes, suggesting potential utility for real-time monitoring. To improve temporal alignment, we also introduced $\Delta T_{\text{dyn}}$ , which incorporates both the trend and variability of ΔT while preserving physiological interpretability.

Given the very limited dataset, we further implemented a data augmentation strategy combining autoregressive modeling of ΔT with transfer-function mapping to NRS, generating one hundred synthetic trajectories per patient. Analyses of these augmented signals revealed consistent lead–lag patterns, correlations, and Granger causality relationships. Raw ΔT frequently led NRS, highlighting its role as an anticipatory biomarker of nociceptive activation, whereas $\Delta T_{\text{dyn}}$ reduced lag and achieved closer synchrony with reported pain, making it suitable for real-time tracking.

Our findings indicate that ΔT and $\Delta T_{\text{dyn}}$ serve as potential indicators of nociception dynamics: the former as an early-warning predictor of nociception, and the latter as a temporally aligned marker of subjective perception. This dual role suggests potential applications in postoperative care, intensive care units, and adaptive analgesia. Importantly, the Anspec-Pro device emerges as a promising non-invasive and practical tool for impedance-based nociceptive monitoring, supporting the integration of physiologically grounded indices into future clinical pain assessment workflows.

## 6 Synthetic patient generation

To expand the limited dataset, we generated one hundred synthetic trajectories for each real patient. The augmentation follows two steps: (i) an autoregressive model of order one (AR(1)) is fitted to the patient’s ΔT sequence and used to generate new ΔT trajectories by resampling noise, and (ii) a patient-specific transfer function is applied to map each synthetic ΔT sequence to a corresponding NRS trajectory. In this way, one hundred synthetic patients are created per real subject. The procedure is summarized in Algorithm 1.

## Data Availability

The original contributions presented in the study are included in the article/supplementary material, further inquiries can be directed to the corresponding author.

## Footnotes

1 Calcium ions (Ca2+) entering the neuron trigger the release of neurotransmitters stored in vesicles.

2 Glutamate is the primary excitatory neurotransmitter in the nervous system, playing a key role in transmitting pain signals.

3 Substance P is a neuropeptide involved in the transmission of pain and other sensory signals, often associated with inflammatory responses.

4 NMDA receptors are ligand-gated ion channels that require both glutamate binding and membrane depolarization to relieve a voltage-dependent magnesium block. Once open, they allow calcium (Ca2+) influx, contributing to long-lasting synaptic excitation.

5 PAD is a mild depolarization of the nociceptor terminal caused by ${\text{GABA}}_{A}$ receptor activation. It paradoxically reduces neurotransmitter release by inactivating voltage-gated sodium channels and decreasing action potential amplitude.

6 These are two-pore domain K^+^ (K2P) channels that remain open at rest and stabilize the membrane potential, making neurons less responsive to excitatory input.

7 Second-order pathways refer to projection neurons in the dorsal horn that relay nociceptive signals to higher brain regions via ascending tracts such as the spinothalamic and spinoreticular pathways. These neurons form the second step in the central processing of pain, following the primary afferent input.

8 Thalamo-cortical networks refer to reciprocal neural connections between the thalamus and various cortical regions. These circuits are essential for processing and relaying sensory information, including nociceptive input.

9 The anterior cingulate cortex (ACC) is a part of the limbic system involved in the affective and attentional dimensions of pain. It plays a critical role in emotional processing, cognitive control, and the evaluation of pain salience.

10 The insular cortex (insula) is involved in interoceptive awareness and the subjective experience of pain. It integrates sensory, affective, and autonomic information and is considered a key hub in pain perception.

11 The prefrontal cortex (PFC) is responsible for higher-order cognitive functions such as decision-making, attention, and emotional regulation. In the context of pain, it contributes to the evaluation, modulation, and behavioral response to noxious stimuli.
